# (*E*)-*N*,*N*-Diethyl-4-{[(4-meth­oxy­phen­yl)imino]­meth­yl}aniline: crystal structure, Hirshfeld surface analysis and energy framework

**DOI:** 10.1107/S2056989024000574

**Published:** 2024-01-26

**Authors:** A. Subashini, R. Kumaravel, B. Tharmalingam, K. Ramamurthi, Aurélien Crochet, Helen Stoeckli-Evans

**Affiliations:** a PG and Research Department of Physics, Srimad Andavan Arts and Science College (Autonomous), Affiliated to Bharathidasan University, Tiruchirappalli 620005, Tamilnadu, India; b Department of Physics, Annapoorana Engineering College (Autonomous), Salem 636308, Tamilnadu, India; c Department of Chemistry, Bharathiyar University, Coimbatore 600 046, Tamilnadu, India; d Crystal Growth and Thin Film Laboratory, Department of Physics, Bharathidasan University, Tiruchirappalli 620024, Tamilnadu, India; e Chemistry Department, University of Fribourg, Chemin du Musee 9, CH-1700 Fribourg, Switzerland; f Institute of Physics, University of Neuchâtel, Rue Emile-Argand 11, CH-2000 Neuchâtel, Switzerland; University of Aberdeen, United Kingdom

**Keywords:** crystal structure, benzyl­ideneaniline, Schiff base, Hirshfeld surface analysis, energy framework

## Abstract

In the title benzyl­ideneaniline Schiff base, C_18_H_22_N_2_O, the aromatic rings are inclined to each other by 46.01 (6)°, while the C_ar_—N= C—C_ar_ torsion angle is 176.9 (1)°. In the crystal, the only identifiable directional inter­action is a weak C—H⋯π hydrogen bond, which generates inversion dimers that stack along the *a*-axis direction.

## Abstract 2

In the title com­pound, a benzyl­ideneaniline Schiff base, the planes of the *p*-substituted aromatic rings subtend a dihedral angle of 46.01 (6)°.

## 1. Chemical context

Schiff bases are known for their distinctive azomethine group (–N=CH–) and ease of synthesis, often by a simple condensation reaction. Brodowska & Łodyga-Chruścińska (2014 ▸, and references therein) have reviewed Schiff bases, covering their biological, anti­bacterial, anti­tfungal, biocidal, anti­malarial and anti­cancer activities, together with their uses in technology, synthesis and chemical analysis. The –N=CH– group plays an important role in forming stable metal com­plexes (Iqbal *et al.*, 1995 ▸), and recently Boulechfar *et al.* (2023 ▸) have reviewed the history, synthesis and applications of Schiff bases and their metal com­plexes. 
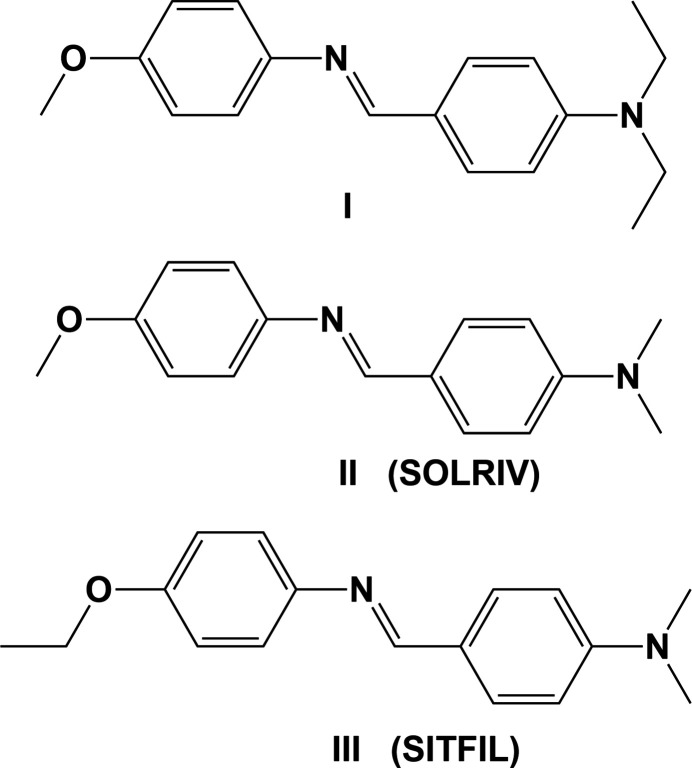


In the solid state, benzyl­ideneanilines adopt a nonplanar conformation, disrupting the π-electron conjugation within the mol­ecule (Bürgi & Dunitz, 1970 ▸). Beyond their chemical properties, benzyl­ideneanilines find practical uses in various applications, such as plaque imaging, as anti-inflammatory agents, and in opto-electronic devices (Lee *et al.*, 2009 ▸; Weszka *et al.*, 2008 ▸; Rodrigues *et al.*, 2003 ▸), and as anti­oxidants (Sunil *et al.*, 2021 ▸).

Herein, we describe the synthesis and crystal structure of the title benzyl­ideneaniline Schiff base (*E*)-*N*,*N*-diethyl-4-{[(4-meth­oxy­phen­yl)imino]­meth­yl}aniline (**I**) and com­pare its structure and Hirshfeld surface to those of related com­pounds.

## 2. Structural commentary

**Figure 1:**
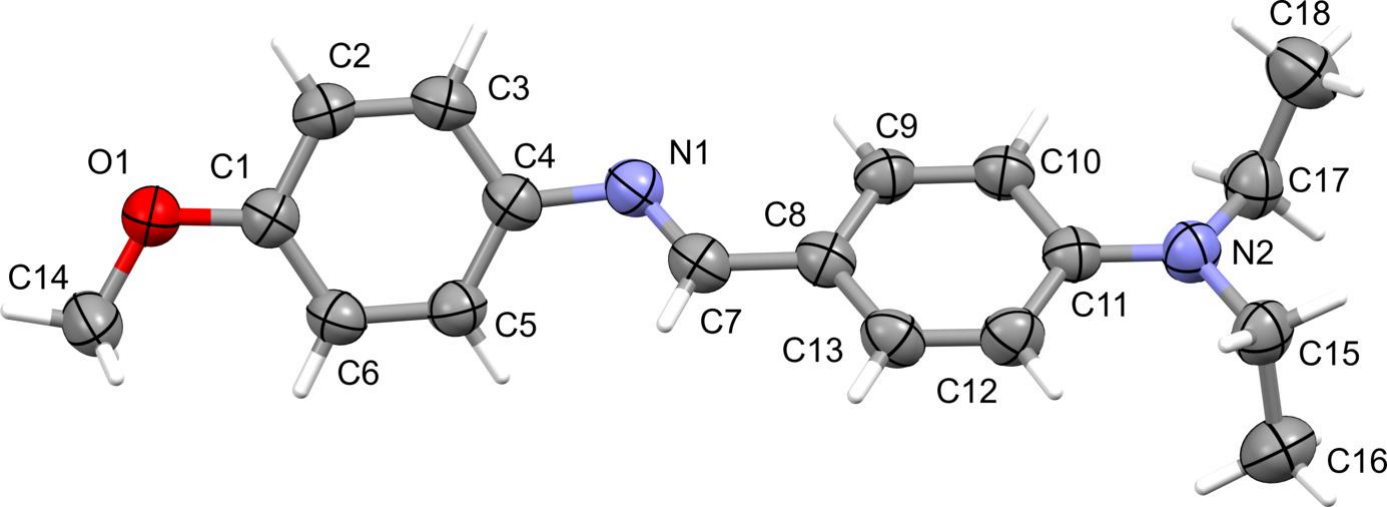
A view of the mol­ecular structure of **I**, with the atom labelling. The displacement ellipsoids are drawn at the 50% probability level.

The title com­pound crystallizes in the triclinic space group *P* 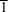 with one mol­ecule in the asymmetric unit (Fig. 1 ▸). The aromatic rings (A = C1–C6 and B = C8–C13) are inclined to each other by 46.01 (6)°, while the C4—N1—C7—C8 torsion angle is 176.9 (1)°. The configuration about the N1=C7 bond is *E* and its bond length is 1.2754 (15) Å. The major twist in the mol­ecule occurs about the C4—N1 bond, as indicated by the C5—C4—N1—C7 torsion angle of −41.89 (16)°. Atom C14 of the meth­oxy group lies almost in the plane of its attached ring [deviation = −0.012 (1) Å]. The N2/C15/C17 moiety is twisted by 12.85 (12)° from its attached ring and the C atom of the C16 methyl group is displaced from the C8–C13 ring by 1.329 (2) Å and C18 is displaced in the opposite sense, by −0.893 (2) Å, which we term a *trans* arrangement (see *Database survey* section).

## 3. Supra­molecular features

**Figure 2:**
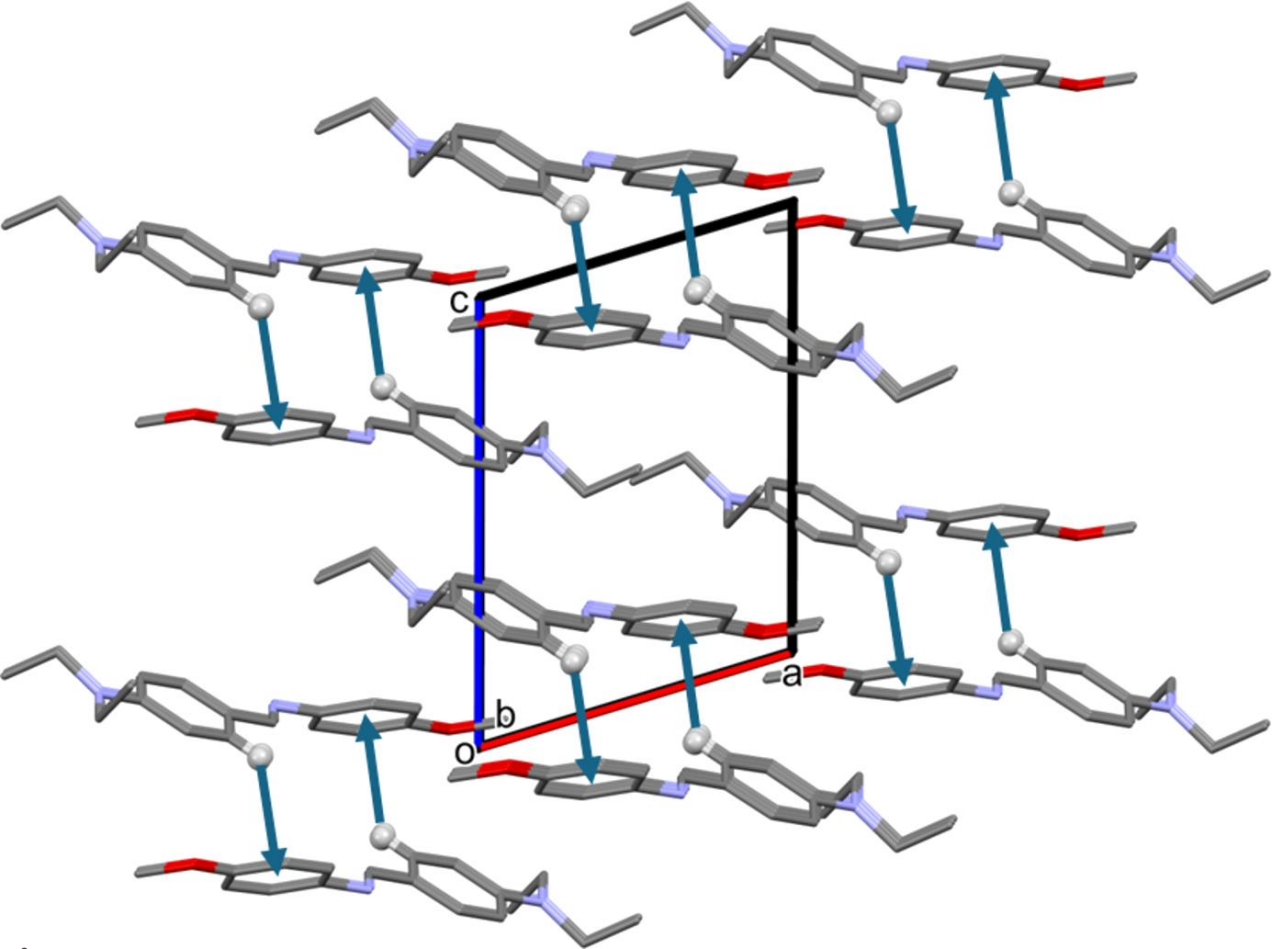
A view along the *b* axis of the crystal packing of **I**. The C—H⋯π inter­actions are indicated by blue arrows (see Table 1 ▸). Only the H atoms involved in these inter­actions have been included.

In the crystal of **I**, the shortest contact involves a pair of very weak C—H⋯π inter­actions (Table 1 ▸). They link inversion-related mol­ecules to form dimers that stack along the *a*-axis direction (Fig. 2 ▸).

## 4. Database survey

A search of the Cambridge Structural Database (CSD, Version 5.44, last update September 2023; Groom *et al.*, 2016 ▸) revealed the presence of two benzyl­ideneaniline Schiff bases similar to **I**, namely, (*E*)-4-{[(4-meth­oxy­phen­yl)imino]­meth­yl}-*N*,*N*-di­methyl­aniline (**II**) (CSD refcode SOLRIV; Sundararaman *et al.*, 2009 ▸) and (*E*)-4-{[(4-eth­oxy­phen­yl)imino]­meth­yl}-*N*,*N*-di­methyl­aniline (**III**) (SITFIL; Wang & Wang, 2008 ▸).

Compound (**II**) crystallizes in the space group *P*2_1_/*n* with two independent mol­ecules in the asymmetric unit. Here, the dihedral angles A/B and A′/B′ are significantly different to each other and to that in com­pound **I**, *viz*. 8.20 (5) and 12.52 (6)°, com­pared to 46.01 (6)° in **I**. The N=C bond lengths are 1.2758 (15) and 1.2731 (16) Å, similar to the value observed for **I**. The C_ar_—N=C—C_ar_ torsion angles are −177.6 (1) and −179.3 (1)°, com­pared to 176.9 (1)° in **I**. In **III**, the aromatic rings are inclined to each other by 61.94 (15)°, while the torsion angle C_ar_—N=C—C_ar_ is 179.3 (3)° and its bond length is 1.269 (4) Å.

A full search of the CSD for *p*-substituted benzyl­ideneanilines gave 229 hits for entries that fitted the following criteria: three-dimensional coordinates available, *R* ≤ 0.075, no disorder, no errors, no polymers, no ions, organics only and only single crystal analyses. An analysis using *Mercury* (Macrae *et al.*, 2020 ▸) of the dihedral angle A/B indicated that it can vary from 0.9° for (*E*)-4-{4-[(4-chloro­benzyl­idene)amino]­benz­yl}oxazolidin-2-one (FORYIX; Kumari *et al.*, 2019 ▸) to 73.4° for 4-[(*E*)-({4-[(4-amino­phen­yl)sulfon­yl]phen­yl}imino)­meth­yl]phenol ethanol solvate (PAWMUX; Afzal *et al.*, 2012 ▸). There are two small clusters grouped around *ca* 6.3 and 51.6°. Compound **II** fits into the first cluster, whereas com­pounds **I** and **III** clearly fit into the second cluster.

The analysis of the N=C bond length indicates that it varies from 1.216 Å for 4-{[4-(di-*p*-tolyl­amino)­benzyl­idene]amino}­benzo­nitrile (JIDRAT; Sun *et al.*, 2023 ▸) to 1.315 Å for (*E*)-4-[4-(di­ethyl­amino)­benzyl­idene­ammonio]­benzene­sulfonate (XAYSOH; Ruanwas *et al.*, 2012 ▸), with a mean value of 1.269 Å [mean deviation of 0.013 Å, skewness −0.162; *Mercury* (Macrae *et al.*, 2020 ▸)]. The C=N bond lengths in **I**, **II** and **III** all fall within the limits indicated from the analysis in *Mercury*.

Another structural feature of com­pound **I** is the arrangement of the ethyl groups of the –N(C_2_H_5_)_2_ moiety. Here, they have a *trans* arrangement with one CH_3_ group directed above the plane of the –CH_2_—N—CH_2_– unit and the other below (Fig. 1 ▸). A search of the CSD for benzyl­ideneanilines with an *N*,*N*-di­ethyl­aniline group gave 12 hits. In nine of these structures the arrangement of this group was the same as that of com­pound **I**, but for three hits an alternative arrangement was found, *viz*. a *cis* arrangement with both CH_3_ groups directed to the same side of the plane of the –CH_2_—N—CH_2_– unit. For example, in 4-chloro-*N*-[4-(di­ethyl­amino)­benzyl­idene]aniline (DUNNAC; Zhang, 2010 ▸), which crystallizes with two independent mol­ecules in the asymmetric unit, both mol­ecules have the *cis* arrangement [Fig. S1(*a*) of the supporting information]. In the 4-bromo derivative, 4-bromo-*N*-[4-(di­ethyl­amino)­benzyl­idene]aniline (SABPOC; Li, 2010 ▸), which also crystallizes with two independent mol­ecules in the asymmetric unit, both arrangements are observed; *i.e.* one *trans* and the other *cis* [Fig. S1(*b*) of the supporting information]. For 4-{[4-(di­ethyl­amino)­benzyl­idene]amino}­benzoic acid, two triclinic polymorphs have been reported, with both structures having two independent mol­ecules in the asymmetric unit. In the first (PUSMUN; Han *et al.*, 2016 ▸), both mol­ecules have a *cis* arrangement, while in the second polymorph (PUSMUN01; Xochicale-Santana *et al.*, 2021 ▸), both mol­ecules have a *trans* arrangement.

**Figure 3:**
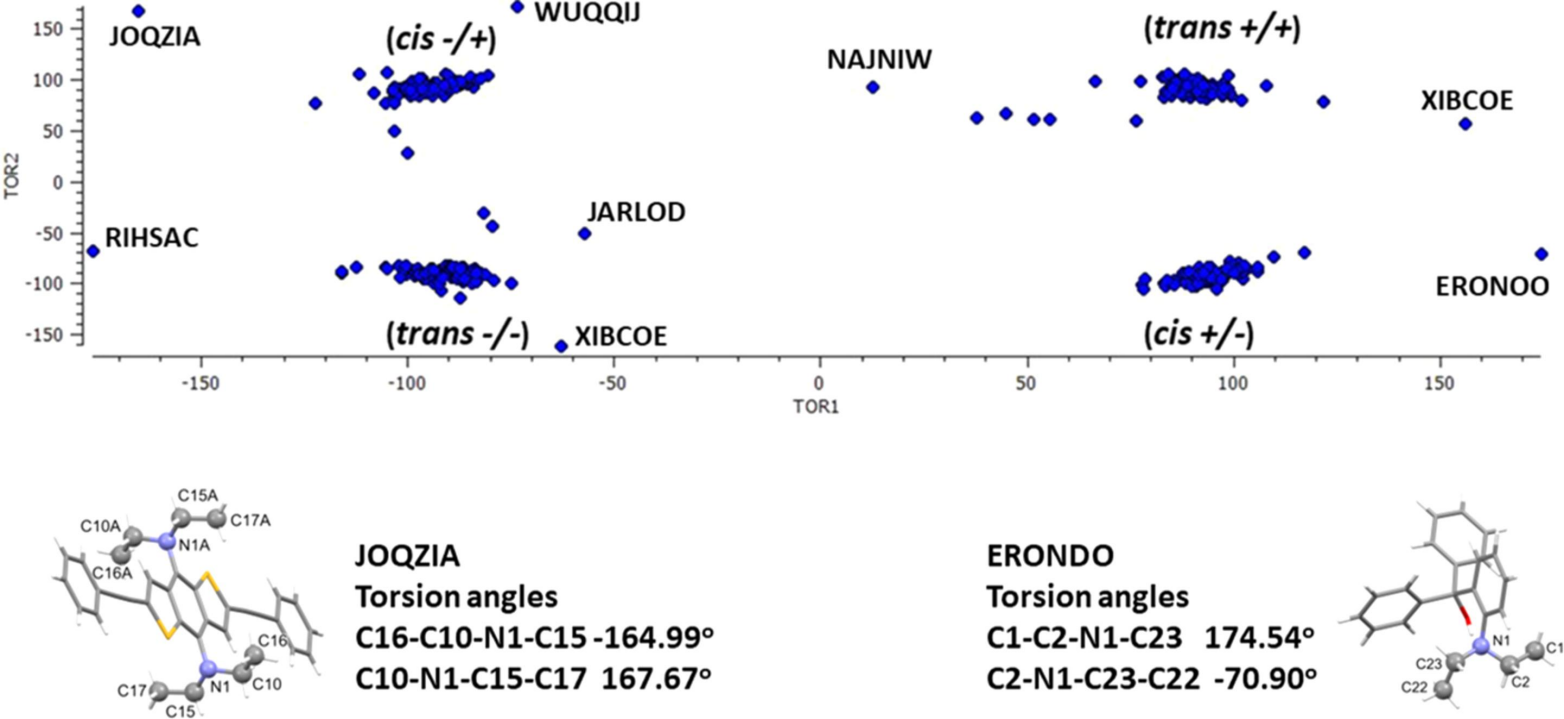
Scatter plot of the CH_2_—N—CH_2_—CH_3_ torsion angles in di­ethyl­amino­benzene derivatives, and the structures of *N*,*N*,*N*′,*N′*-tetra­ethyl-2,6-bis­(phenyl­ethyn­yl)thieno[2,3-*f*][1]benzo­thio­phene-4,8-di­amine (JOQZIA; Wen *et al.*, 2015 ▸) and 2-di­ethyl­amino­phen­yl)di­phenyl­methanol (ERONDO; Al-Masri *et al.*, 2004 ▸).

A more extensive search for di­ethyl­amino­benzene derivatives gave over 300 hits for structures with the same search criteria as above. An analysis of the two CH_3_—CH_2_—N—CH_2_ torsion angles is shown in a scatter plot (Fig. 3 ▸). It can be seen that the majority of com­pounds have either the *cis* (−/+ or +/−) or the *trans* (+/+ or −/−) arrangement. Some of the outliers indicate an inter­mediate state with one large torsion angle and the other quite small, for example, (2-di­ethyl­amino­phen­yl)di­phenyl­methanol (ERONDO; Al-Masri *et al.*, 2004 ▸), whose structure is illustrated in Fig. 3 ▸. Finally, in one com­pound, *viz. N*,*N*,*N*′,*N′*-tetra­ethyl-2,6-bis­(phenyl­ethyn­yl)thieno[2,3-*f*][1]benzo­thio­phene-4,8-di­amine (JOQZIA; Wen *et al.*, 2015 ▸), a unique arrangement was observed with both ethyl groups having an extended conformation (see Fig. 3 ▸).

## 5. Hirshfeld surface analysis and two-dimensional fingerprint plots

**Figure 4:**
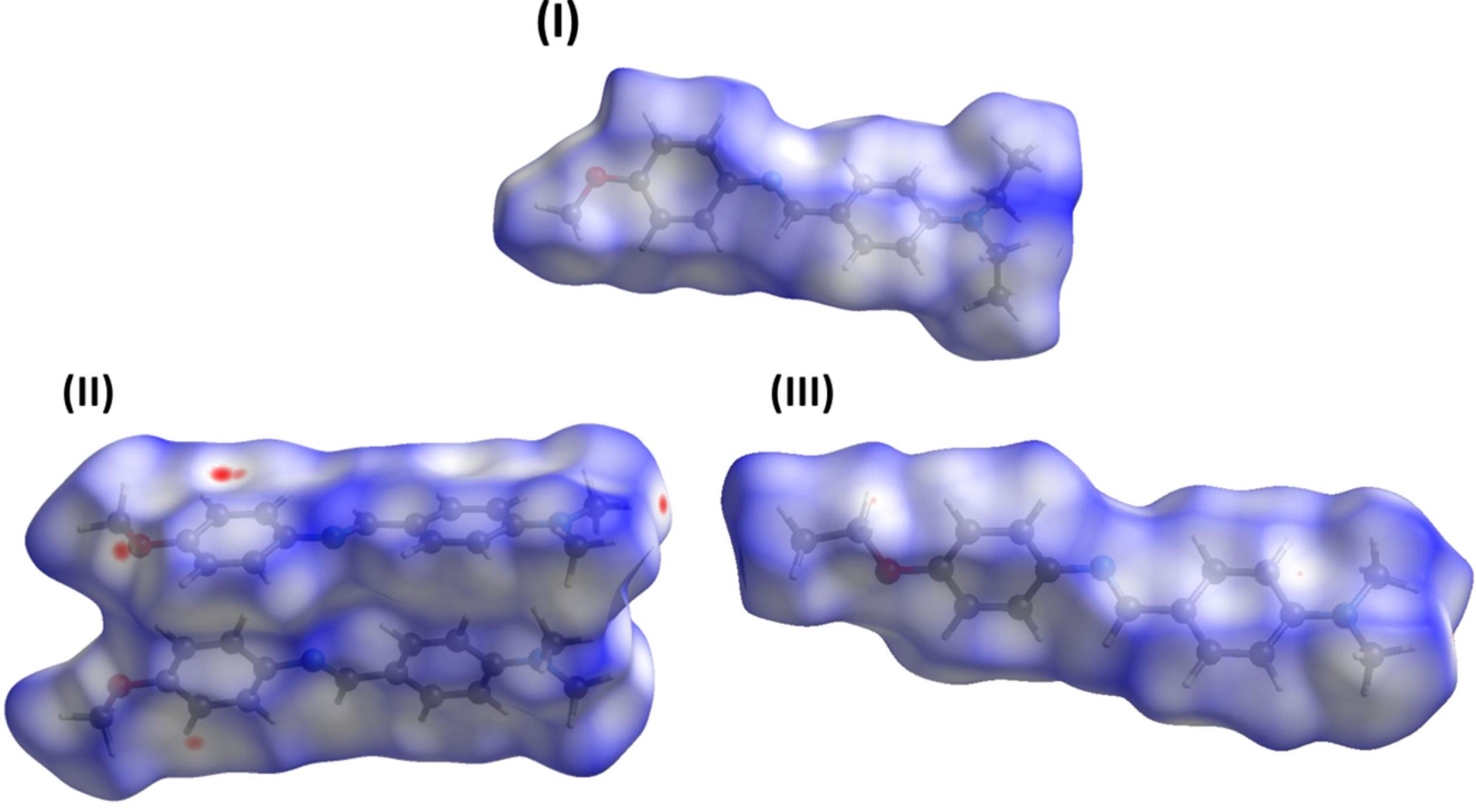
The Hirshfeld surfaces of com­pounds (*a*) **I**, (*b*) **II** and (*c*) **III**, mapped over *d*_norm_ in the colour ranges of 0.00 to 1.41, −0.08 to 1.26 and −0.02 to 1.22 a.u., respectively.

The Hirshfeld surface (HS) analyses and the associated two-dimensional fingerprint plots were performed with *CrystalExplorer17* (Spackman *et al.*, 2021 ▸) following the protocol of Tan *et al.* (2019 ▸). The Hirshfeld surfaces for com­pounds **I**, **II** and **III** are com­pared in Fig. 4 ▸. The absence of promient red spots indicate that short contacts are not particularly significant in the packing of the three com­pounds. The short contacts in the crystals of the three com­pounds are com­pared in Table S1 of the supporting information. It is not surprising that for **II**, with a total of seven C—H⋯π inter­actions in the crystal (Sundararaman *et al.*, 2009 ▸), that there are a large number of C⋯H contacts.

**Figure 5:**
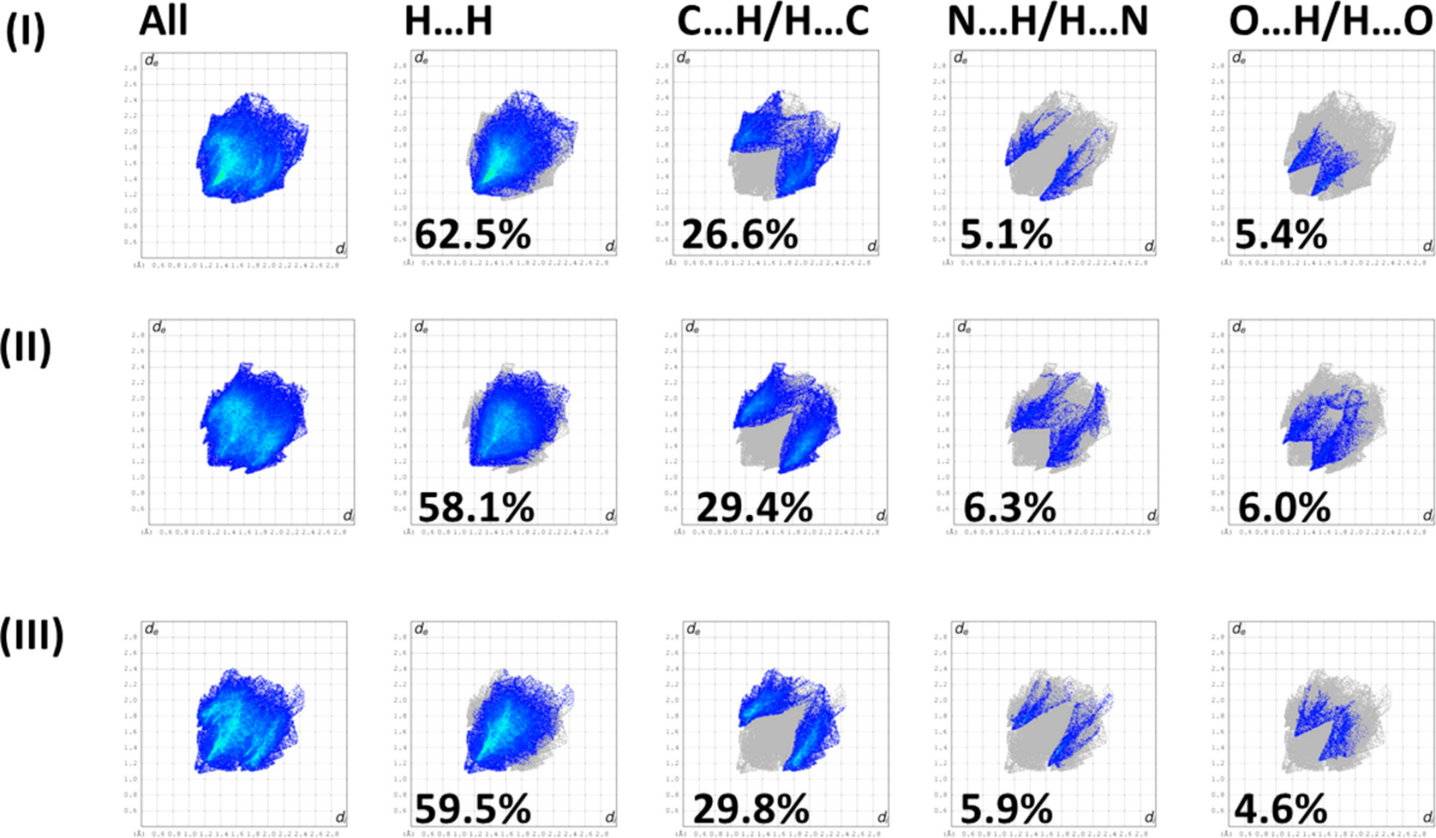
The full two-dimensional fingerprint plots for com­pounds (*a*) **I**, (*b*) **II** and (*c*) **III**, and those delineated into H⋯H, C⋯H/H⋯C, N⋯H/H⋯N and O⋯H/H⋯O contacts.

The full two-dimensional fingerprint plots for **I**, **II** and **III** are given in Fig. 5 ▸. The contributions of the various inter­atomic contacts to the Hirshfeld surfaces for the three com­pounds are com­pared in Table 2 ▸. In all three com­pounds, the H⋯H contacts have a major contribution, *i.e.* 62.5% for **I**, 58.1% for the two independent mol­ecule of **II** and 59.5% for **III**. The second most significant contributions are from the C⋯H/H⋯C contacts, 26.6, 29.4 and 29.8%, respectively, reflecting the presence of C—H⋯π inter­actions present in all three crystal structures. The other inter­atomic contacts, such as the N⋯H/H⋯N contacts, contribute from 5.1 to 6.3%, and the O⋯H/H⋯O contacts contribute from 4.6 to 6.0%. The C⋯C or O⋯O contacts contribute less than 1%.

## 6. Energy frameworks

**Figure 6:**
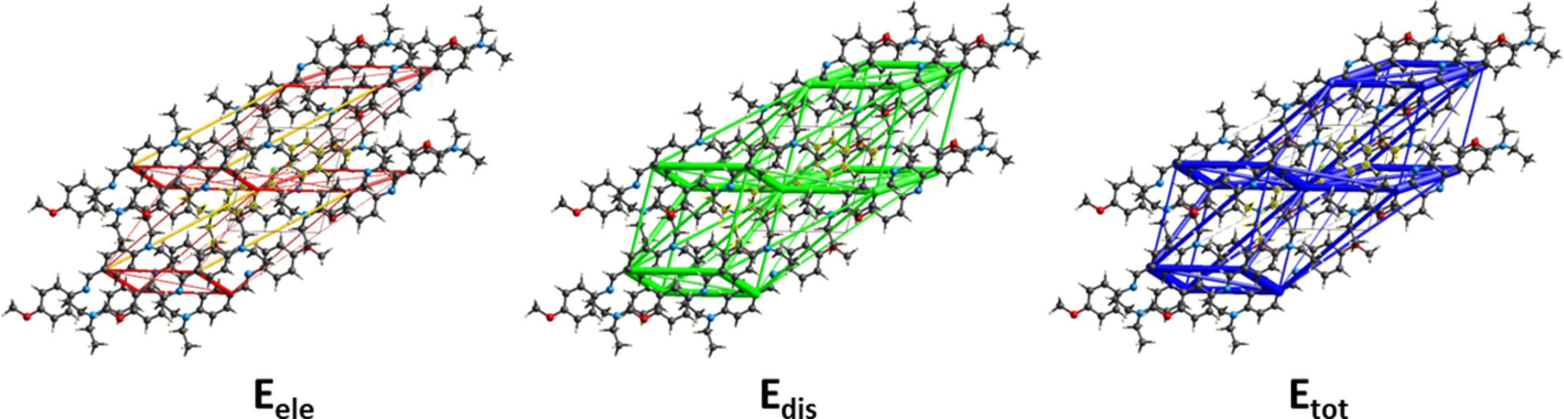
The energy frameworks calculated for **I**, viewed along the *b*-axis direction, showing the electrostatic potential forces (*E*_ele_), the dispersion forces (*E*_dis_) and the total energy diagrams (*E*_tot_).

A com­parison of the energy frameworks calculated for **I**, showing the electrostatic potential forces (*E*_ele_), the dispersion forces (*E*_dis_) and the total energy diagrams (*E*_tot_), are shown in Fig. 6 ▸. Those for com­pounds **II** and **III** are given, respectively, in Figs. S3 and S4 of the supporting information. The energies were obtained by using wave functions at the HF/3-2IG level of theory. The cylindrical radii are proportional to the relative strength of the corresponding energies (Spackman *et al.*, 2021 ▸; Tan *et al.*, 2019 ▸). They have been adjusted to the same scale factor of 90 with a cut-off value of 6 kJ mol^−1^ within a radius of 3.8 Å of a central reference mol­ecule.

For all three com­pounds, the major contribution to the inter­molecular inter­actions is from dispersion forces (*E*_dis_), reflecting the absence of C—H⋯O or C—H⋯N hydrogen bonds in the crystals. The colour-coded inter­action mappings within a radius of 3.8 Å of a central reference mol­ecule and the various contributions to the total energy (*E*_tot_) for com­pounds **I**, **II** and **III** are given in Figs. S5, S6 and S7, respectively, of the supporting information.

## 7. Synthesis and crystallization

Compound **I** was synthesized by condensing *p*-di­ethyl­amino­benzaldehyde and *p*-meth­oxy­aniline (1:1) dissolved in methanol. The reaction mixture was heated under reflux for 6 h at ∼363 K and then cooled to room temperature. The precipitated product was dissolved in methanol. Yellow prismatic single crystals of **I** were obtained by slow evaporation of the solvent at room temperature over a period of *ca* 15 d.

A Shimadzu IR Affinity-1 Fourier transform infrared (FT–IR) spectrometer was used to record the FT–IR spectrum of **I** using the KBr pellet technique in the range 400–4000 cm^−1^ (Fig. S8 of the supporting information). The absorption band at 1603 cm^−1^ confirms the formation of the C=N groups. The aromatic ring C=C stretching vibrations are observed in the range 1468–1585 cm^−1^. The aromatic C—H in-plane bending modes are observed in the region 1005–1292 cm^−1^, whereas the out-of-plane bending modes are observed in the range 762–973 cm^−1^.

The ^1^H and ^13^C nuclear magnetic resonance (NMR) spectra of com­pound **I** (Fig. S9 of the supporting information) were recorded using a Bruker Advance Neo 400 MHz NMR spectrometer. Deuterated chloro­form (CDCl_3_-*d*) was employed as the solvent, with tetra­methyl­silane (TMS) serving as the inter­nal standard. In the ^1^H NMR spectrum of **I**, the singlet peak at 8.30 ppm is attributed to the azomethine (–N=CH–) proton, while signals observed at 7.73, 7.18, 7.16 and 6.89 ppm are attributed to the aromatic protons. Additionally, there are sharp singlet peaks at 3.80 ppm, corresponding to the meth­oxy protons (O—CH_3_). The protons of the di­ethyl­amino group were detected at 1.19 ppm as a triplet (CH_3_) and at 3.41 ppm as a quartet (CH_2_). In the ^13^C NMR spectrum of **I**, the resonance at 158.70 ppm signifies the presence of the azomethine (–N=CH–) unit, 55.51 ppm is associated with the CH_3_—O group, 44.51 ppm is related to the methyl­ene C atoms of the (CH_3_CH_2_)_2_—N group and 12.62 ppm corresponds to the methyl C atoms of the (CH_3_CH_2_)_2_—N group.

An SDT Q600 V20.9 Build 20 TA instrument were used to measure the thermogravimetric analysis (TGA) and the differential thermal analysis (DTA) in the temperature range 303–723 K (Fig. S10 of the supporting information) with a heating rate of 20 K min^−1^. A small peak observed at ∼377 K (Fig. S10) in the DTA curve corresponds to the melting point of the material. The material is stable up to 483 K, after which it starts to decom­pose.

## 8. Refinement

Crystal data, data collection and structure refinement details are summarized in Table 3 ▸. The C-bound H atoms were included in calculated positions and treated as riding atoms, with C—H = 0.94–0.98 Å and *U*_iso_(H) = 1.5*U*_eq_(C) for methyl H atoms and 1.2*U*_eq_(C) for other H atoms.

## Supplementary Material

Crystal structure: contains datablock(s) I, Global. DOI: 10.1107/S2056989024000574/hb8091sup1.cif

Structure factors: contains datablock(s) I. DOI: 10.1107/S2056989024000574/hb8091Isup2.hkl

Table S1. Short contacts in the crystal structures of com­pounds I, II and III. Figs. S1-S10. Energy frameworks and FTIR, NMR and DSC and TGA data. DOI: 10.1107/S2056989024000574/hb8091sup3.pdf

Supporting information file. DOI: 10.1107/S2056989024000574/hb8091Isup4.cml

CCDC reference: 2325829

Additional supporting information:  crystallographic information; 3D view; checkCIF report

## Figures and Tables

**Table 1:** Hydrogen-bond geometry (Å, °) *Cg*1 is the centroid of the C1–C6 ring.

*D*—H⋯*A*	*D*—H	H⋯*A*	*D*⋯*A*	*D*—H⋯*A*
C13—H13⋯*Cg*1^i^	0.94	2.98	3.659 (1)	130

Symmetry code: (i) 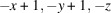 .

**Table 2:** Relative percentage contributions of close contacts to the Hirshfeld surfaces of com­pounds **I**, **II** and **III** **II*a*** and **II*b*** refer to the two independent mol­ecules of com­pound **II**.

Contact	**I**	**II**	**II*a***	**II*b***	**III**
H⋯H	62.5	58.1	53.9	55.2	59.5
C⋯H/H⋯C	26.6	29.4	34.3	32.0	29.8
N⋯H/H⋯N	5.1	6.3	5.6	6.5	5.9
O⋯H/H⋯O	5.4	6.0	6.0	6.2	4.6

**Table 3:** Experimental details

Crystal data	
Chemical formula	C_18_H_22_N_2_O
*M*_r_	282.37
Crystal system, space group	Triclinic, *P* 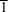
Temperature (K)	250
*a*, *b*, *c* (Å)	8.3830 (7), 9.2872 (7), 11.2981 (9)
α, β, γ (°)	78.991 (6), 71.009 (6), 74.174 (6)
*V* (Å^3^)	795.14 (12)
*Z*	2
Radiation type	Mo *K*α
μ (mm^−1^)	0.07
Crystal size (mm)	0.68 × 0.47 × 0.28

Data collection	
Diffractometer	STOE IPDS II
Absorption correction	Multi-scan [*X-RED32* (Stoe & Cie, 2018 ▸) and *X-AREA LANA* (Stoe & Cie, 2018 ▸)]
*T*_min_, *T*_max_	0.697, 0.989
No. of measured, independent and observed [*I* > 2σ(*I*)] reflections	11520, 3183, 2453
*R*_int_	0.030
(sin θ/λ)_max_ (Å^−1^)	0.622

Refinement	
*R*[*F* ^2^ > 2σ(*F* ^2^)], *wR*(*F* ^2^), *S*	0.035, 0.100, 1.04
No. of reflections	3183
No. of parameters	194
H-atom treatment	H-atom parameters constrained
Δρ_max_, Δρ_min_ (e Å^−3^)	0.11, −0.10

Computer programs: *X-AREA* , *X-RED32* and *X-AREA LANA* (Stoe & Cie, 2018 ▸), *SHELXT2014* (Sheldrick, 2015*a* ▸), *PLATON* (Spek, 2020 ▸), *Mercury* (Macrae *et al.*, 2020 ▸), *SHELXL2018* (Sheldrick, 2015*b* ▸) and *publCIF* (Westrip, 2010 ▸).

## supplementary crystallographic information

### Crystal data

**Table:**

C_18_H_22_N_2_O	*Z* = 2
*M**_r_* = 282.37	*F*(000) = 304
Triclinic, *P*1	*D*_x_ = 1.179 Mg m^−^^3^
*a* = 8.3830 (7) Å	Mo *K*α radiation, λ = 0.71073 Å
*b* = 9.2872 (7) Å	Cell parameters from 8317 reflections
*c* = 11.2981 (9) Å	θ = 1.9–26.6°
α = 78.991 (6)°	µ = 0.07 mm^−^^1^
β = 71.009 (6)°	*T* = 250 K
γ = 74.174 (6)°	Prism, yellow
*V* = 795.14 (12) Å^3^	0.68 × 0.47 × 0.28 mm

### Data collection

**Table:**

STOE IPDS II diffractometer	3183 independent reflections
Radiation source: sealed X-ray tube, 12 x 0.4 mm long-fine focus	2453 reflections with *I* > 2σ(*I*)
Plane graphite monochromator	*R*_int_ = 0.030
Detector resolution: 6.67 pixels mm^-1^	θ_max_ = 26.2°, θ_min_ = 1.9°
rotation method, ω scans	*h* = −10→10
Absorption correction: multi-scan [*X-RED32* (Stoe & Cie, 2018) and *X-AREA LANA* (Stoe & Cie, 2018)]	*k* = −11→11
*T*_min_ = 0.697, *T*_max_ = 0.989	*l* = −13→14
11520 measured reflections	

### Refinement

**Table:**

Refinement on *F*^2^	Secondary atom site location: difference Fourier map
Least-squares matrix: full	Hydrogen site location: inferred from neighbouring sites
*R*[*F*^2^ > 2σ(*F*^2^)] = 0.035	H-atom parameters constrained
*wR*(*F*^2^) = 0.100	*w* = 1/[σ^2^(*F*_o_^2^) + (0.0477*P*)^2^ + 0.076*P*] where *P* = (*F*_o_^2^ + 2*F*_c_^2^)/3
*S* = 1.04	(Δ/σ)_max_ < 0.001
3183 reflections	Δρ_max_ = 0.11 e Å^−^^3^
194 parameters	Δρ_min_ = −0.10 e Å^−^^3^
0 restraints	Extinction correction: (SHELXL2018; Sheldrick, 2015b), Fc^*^=kFc[1+0.001xFc^2^λ^3^/sin(2θ)]^-1/4^
Primary atom site location: dual	Extinction coefficient: 0.06 (1)

### Special details

**Table:**

Geometry. All esds (except the esd in the dihedral angle between two l.s. planes) are estimated using the full covariance matrix. The cell esds are taken into account individually in the estimation of esds in distances, angles and torsion angles; correlations between esds in cell parameters are only used when they are defined by crystal symmetry. An approximate (isotropic) treatment of cell esds is used for estimating esds involving l.s. planes.

### Fractional atomic coordinates and isotropic or equivalent isotropic displacement parameters (Å^2^)

**Table:**

	*x*	*y*	*z*	*U*_iso_*/*U*_eq_	
O1	0.90654 (10)	0.86089 (9)	0.06035 (8)	0.0546 (2)	
N1	0.34566 (12)	0.58694 (11)	0.23619 (9)	0.0508 (3)	
N2	−0.20580 (13)	0.16536 (11)	0.38486 (9)	0.0527 (3)	
C1	0.77767 (14)	0.78348 (12)	0.10357 (10)	0.0454 (3)	
C2	0.61011 (15)	0.87089 (12)	0.12118 (11)	0.0502 (3)	
H2	0.591811	0.976243	0.103921	0.060*	
C3	0.47095 (15)	0.80443 (13)	0.16366 (11)	0.0513 (3)	
H3	0.358218	0.865153	0.176449	0.062*	
C4	0.49434 (14)	0.64815 (12)	0.18812 (10)	0.0462 (3)	
C5	0.66201 (14)	0.56221 (12)	0.17208 (11)	0.0490 (3)	
H5	0.680241	0.456927	0.190073	0.059*	
C6	0.80332 (14)	0.62806 (12)	0.13011 (11)	0.0486 (3)	
H6	0.915970	0.567772	0.119672	0.058*	
C7	0.34757 (14)	0.46457 (13)	0.19969 (10)	0.0491 (3)	
H7	0.446601	0.421105	0.138215	0.059*	
C8	0.20522 (14)	0.38883 (12)	0.24788 (10)	0.0459 (3)	
C9	0.05732 (14)	0.43984 (12)	0.34505 (10)	0.0470 (3)	
H9	0.049892	0.525924	0.380601	0.056*	
C10	−0.07673 (14)	0.36818 (12)	0.38981 (11)	0.0474 (3)	
H10	−0.173924	0.406192	0.455066	0.057*	
C11	−0.07201 (14)	0.23796 (12)	0.33985 (10)	0.0445 (3)	
C12	0.07565 (15)	0.18844 (13)	0.24069 (11)	0.0519 (3)	
H12	0.083149	0.103674	0.203407	0.062*	
C13	0.20913 (15)	0.26207 (13)	0.19749 (11)	0.0520 (3)	
H13	0.306292	0.225458	0.131648	0.062*	
C14	1.07994 (16)	0.77581 (15)	0.04265 (14)	0.0675 (4)	
H14A	1.106339	0.705115	−0.017850	0.101*	
H14B	1.093169	0.721030	0.122330	0.101*	
H14C	1.158592	0.843244	0.011381	0.101*	
C15	−0.20452 (17)	0.03482 (13)	0.33003 (12)	0.0570 (3)	
H15A	−0.153911	0.050389	0.238532	0.068*	
H15B	−0.324234	0.027042	0.346349	0.068*	
C16	−0.1037 (2)	−0.11202 (15)	0.38128 (16)	0.0778 (4)	
H16A	−0.152434	−0.128065	0.471944	0.117*	
H16B	0.016571	−0.107421	0.361391	0.117*	
H16C	−0.110656	−0.194522	0.343312	0.117*	
C17	−0.34025 (16)	0.19415 (14)	0.50416 (11)	0.0563 (3)	
H17A	−0.297730	0.241588	0.554642	0.068*	
H17B	−0.362182	0.097789	0.551020	0.068*	
C18	−0.50769 (18)	0.29363 (18)	0.48748 (15)	0.0770 (4)	
H18A	−0.551352	0.247013	0.438293	0.115*	
H18B	−0.488023	0.390729	0.443997	0.115*	
H18C	−0.591789	0.307371	0.569374	0.115*	

### Atomic displacement parameters (Å^2^)

**Table:**

	*U*^11^	*U*^22^	*U*^33^	*U*^12^	*U*^13^	*U*^23^
O1	0.0506 (5)	0.0498 (5)	0.0639 (5)	−0.0138 (4)	−0.0168 (4)	−0.0036 (4)
N1	0.0456 (6)	0.0515 (6)	0.0536 (6)	−0.0091 (4)	−0.0137 (4)	−0.0055 (4)
N2	0.0531 (6)	0.0540 (6)	0.0532 (6)	−0.0159 (5)	−0.0113 (4)	−0.0133 (4)
C1	0.0484 (6)	0.0478 (6)	0.0415 (6)	−0.0113 (5)	−0.0146 (5)	−0.0054 (5)
C2	0.0542 (7)	0.0418 (6)	0.0536 (7)	−0.0063 (5)	−0.0176 (5)	−0.0057 (5)
C3	0.0449 (6)	0.0499 (6)	0.0549 (7)	−0.0006 (5)	−0.0156 (5)	−0.0090 (5)
C4	0.0447 (6)	0.0500 (6)	0.0429 (6)	−0.0086 (5)	−0.0125 (5)	−0.0063 (5)
C5	0.0498 (7)	0.0429 (6)	0.0522 (6)	−0.0072 (5)	−0.0151 (5)	−0.0045 (5)
C6	0.0433 (6)	0.0478 (6)	0.0514 (6)	−0.0039 (5)	−0.0150 (5)	−0.0053 (5)
C7	0.0447 (6)	0.0547 (7)	0.0457 (6)	−0.0067 (5)	−0.0133 (5)	−0.0065 (5)
C8	0.0458 (6)	0.0494 (6)	0.0424 (6)	−0.0074 (5)	−0.0154 (5)	−0.0052 (5)
C9	0.0508 (7)	0.0436 (6)	0.0475 (6)	−0.0077 (5)	−0.0152 (5)	−0.0099 (5)
C10	0.0463 (6)	0.0469 (6)	0.0457 (6)	−0.0054 (5)	−0.0099 (5)	−0.0110 (5)
C11	0.0463 (6)	0.0452 (6)	0.0435 (6)	−0.0077 (5)	−0.0172 (5)	−0.0052 (5)
C12	0.0559 (7)	0.0516 (6)	0.0503 (6)	−0.0089 (5)	−0.0146 (5)	−0.0169 (5)
C13	0.0473 (6)	0.0591 (7)	0.0466 (6)	−0.0063 (5)	−0.0090 (5)	−0.0155 (5)
C14	0.0482 (7)	0.0648 (8)	0.0862 (10)	−0.0149 (6)	−0.0186 (7)	0.0009 (7)
C15	0.0620 (8)	0.0554 (7)	0.0608 (7)	−0.0179 (6)	−0.0205 (6)	−0.0118 (6)
C16	0.0840 (10)	0.0547 (8)	0.0908 (11)	−0.0109 (7)	−0.0250 (8)	−0.0068 (7)
C17	0.0576 (7)	0.0601 (7)	0.0518 (7)	−0.0212 (6)	−0.0100 (5)	−0.0065 (6)
C18	0.0587 (8)	0.0865 (10)	0.0785 (10)	−0.0084 (7)	−0.0123 (7)	−0.0176 (8)

### Geometric parameters (Å, º)

**Table:**

O1—C1	1.3669 (13)	C9—H9	0.9400
O1—C14	1.4220 (14)	C10—C11	1.4153 (15)
N1—C7	1.2754 (15)	C10—H10	0.9400
N1—C4	1.4140 (14)	C11—C12	1.4081 (16)
N2—C11	1.3716 (14)	C12—C13	1.3756 (16)
N2—C15	1.4592 (14)	C12—H12	0.9400
N2—C17	1.4639 (15)	C13—H13	0.9400
C1—C6	1.3882 (16)	C14—H14A	0.9700
C1—C2	1.3891 (15)	C14—H14B	0.9700
C2—C3	1.3739 (16)	C14—H14C	0.9700
C2—H2	0.9400	C15—C16	1.5158 (19)
C3—C4	1.3958 (16)	C15—H15A	0.9800
C3—H3	0.9400	C15—H15B	0.9800
C4—C5	1.3868 (15)	C16—H16A	0.9700
C5—C6	1.3859 (15)	C16—H16B	0.9700
C5—H5	0.9400	C16—H16C	0.9700
C6—H6	0.9400	C17—C18	1.5025 (19)
C7—C8	1.4505 (16)	C17—H17A	0.9800
C7—H7	0.9400	C17—H17B	0.9800
C8—C13	1.3907 (15)	C18—H18A	0.9700
C8—C9	1.3997 (15)	C18—H18B	0.9700
C9—C10	1.3678 (15)	C18—H18C	0.9700
			
C1—O1—C14	117.48 (9)	C12—C11—C10	116.55 (10)
C7—N1—C4	119.31 (10)	C13—C12—C11	121.08 (10)
C11—N2—C15	121.61 (9)	C13—C12—H12	119.5
C11—N2—C17	122.15 (9)	C11—C12—H12	119.5
C15—N2—C17	115.20 (9)	C12—C13—C8	122.25 (10)
O1—C1—C6	124.99 (10)	C12—C13—H13	118.9
O1—C1—C2	115.67 (9)	C8—C13—H13	118.9
C6—C1—C2	119.35 (10)	O1—C14—H14A	109.5
C3—C2—C1	120.45 (10)	O1—C14—H14B	109.5
C3—C2—H2	119.8	H14A—C14—H14B	109.5
C1—C2—H2	119.8	O1—C14—H14C	109.5
C2—C3—C4	121.04 (10)	H14A—C14—H14C	109.5
C2—C3—H3	119.5	H14B—C14—H14C	109.5
C4—C3—H3	119.5	N2—C15—C16	113.41 (11)
C5—C4—C3	117.97 (10)	N2—C15—H15A	108.9
C5—C4—N1	123.58 (10)	C16—C15—H15A	108.9
C3—C4—N1	118.30 (10)	N2—C15—H15B	108.9
C6—C5—C4	121.49 (10)	C16—C15—H15B	108.9
C6—C5—H5	119.3	H15A—C15—H15B	107.7
C4—C5—H5	119.3	C15—C16—H16A	109.5
C5—C6—C1	119.68 (10)	C15—C16—H16B	109.5
C5—C6—H6	120.2	H16A—C16—H16B	109.5
C1—C6—H6	120.2	C15—C16—H16C	109.5
N1—C7—C8	123.56 (11)	H16A—C16—H16C	109.5
N1—C7—H7	118.2	H16B—C16—H16C	109.5
C8—C7—H7	118.2	N2—C17—C18	113.31 (11)
C13—C8—C9	116.81 (10)	N2—C17—H17A	108.9
C13—C8—C7	120.93 (10)	C18—C17—H17A	108.9
C9—C8—C7	122.25 (10)	N2—C17—H17B	108.9
C10—C9—C8	121.97 (10)	C18—C17—H17B	108.9
C10—C9—H9	119.0	H17A—C17—H17B	107.7
C8—C9—H9	119.0	C17—C18—H18A	109.5
C9—C10—C11	121.32 (10)	C17—C18—H18B	109.5
C9—C10—H10	119.3	H18A—C18—H18B	109.5
C11—C10—H10	119.3	C17—C18—H18C	109.5
N2—C11—C12	121.94 (10)	H18A—C18—H18C	109.5
N2—C11—C10	121.51 (10)	H18B—C18—H18C	109.5
			
C14—O1—C1—C6	−0.78 (16)	C7—C8—C9—C10	179.79 (10)
C14—O1—C1—C2	179.17 (10)	C8—C9—C10—C11	0.15 (17)
O1—C1—C2—C3	179.63 (10)	C15—N2—C11—C12	−1.67 (16)
C6—C1—C2—C3	−0.42 (16)	C17—N2—C11—C12	166.16 (11)
C1—C2—C3—C4	−1.05 (17)	C15—N2—C11—C10	177.40 (10)
C2—C3—C4—C5	2.01 (17)	C17—N2—C11—C10	−14.76 (16)
C2—C3—C4—N1	177.64 (10)	C9—C10—C11—N2	179.70 (10)
C7—N1—C4—C5	−41.89 (16)	C9—C10—C11—C12	−1.18 (16)
C7—N1—C4—C3	142.74 (11)	N2—C11—C12—C13	−179.49 (10)
C3—C4—C5—C6	−1.56 (17)	C10—C11—C12—C13	1.38 (17)
N1—C4—C5—C6	−176.95 (10)	C11—C12—C13—C8	−0.58 (18)
C4—C5—C6—C1	0.15 (17)	C9—C8—C13—C12	−0.49 (17)
O1—C1—C6—C5	−179.19 (10)	C7—C8—C13—C12	−179.59 (11)
C2—C1—C6—C5	0.86 (16)	C11—N2—C15—C16	83.50 (15)
C4—N1—C7—C8	176.85 (10)	C17—N2—C15—C16	−85.13 (14)
N1—C7—C8—C13	174.93 (11)	C11—N2—C17—C18	102.52 (13)
N1—C7—C8—C9	−4.12 (17)	C15—N2—C17—C18	−88.92 (13)
C13—C8—C9—C10	0.70 (16)		

### Hydrogen-bond geometry (Å, º)

*Cg*1 is the centroid of the C1–C6 ring.

**Table:**

*D*—H···*A*	*D*—H	H···*A*	*D*···*A*	*D*—H···*A*
C13—H13···*Cg*1^i^	0.94	2.98	3.659 (1)	130

Symmetry code: (i) −*x*+1, −*y*+1, −*z*.
