# Supplementary material for: (E)-N,N-Diethyl-4-{[(4-meth­oxy­phen­yl)imino]­meth­yl}aniline: crystal structure, Hirshfeld surface analysis and energy framework
Source: Acta Crystallogr E Crystallogr Commun. 2024 Jan 26;80(Pt 2):201–6. doi: 10.1107/S2056989024000574 (PMC10848986; doi:10.1107/S2056989024000574)
Supplement: Supplementary file 3 [file e-80-00201-sup3.pdf]

Table S1. Close contacts (Å) in the crystal structures of compounds I, II and III.

Atom1	Atom2	Length (Å)	Length-VdW (Å)	Symm. op. 1	Symm. op. 2
**I**					
**O1**	H14C	2.669	-0.051	x, y, z	2-x, 2-y, -z
**N1**	H14B	2.722	-0.028	x, y, z	-1+x, y, z
**II**					
**H16B**	C31	2.748	-0.151	x, y, z	x, y, z
**H36C**	O2	2.612	-0.108	x, y, z	1.5-x, -1/2+y, 1.5-z
**H6**	C26	2.802	-0.098	x, y ,z	-1+x, y, z
**C1**	H34A	2.810	-0.090	x, y, z	-1/2+x, 1/2-y, 1/2+z
**H6**	C25	2.822	-0.078	x, y, z	-1+x, y, z
**H3**	C22	2.830	-0.070	x, y, z	x, y, z
**C6**	C34	3.332	-0.068	x, y, z	-1/2+x, 1/2-y, 1/2+z
**O1**	H22	2.693	-0.027	x, y, z	-1/2+x, 1/2-y, -1/2+z
**C6**	H34C	2.885	-0.015	x, y, z	-1/2+x, 1/2-y, 1/2+z
**H2**	O2	2.709	-0.012	x, y, z	x, y, z
**H3**	C21	2.891	-0.009	x, y, z	x, y, z
**C7**	H35B	2.893	-0.007	x, y, z	1-x, -y, 2-z
**C1**	C34	3.397	-0.003	x, y, z	-1/2+x, 1/2-y, 1/2+z
**III**					
**C5**	H17	2.863	-0.037	x, y, z	1-x, -y, 1-z
**H7**	C17	2.866	-0.034	x, y, z	-1+x, y, -2+z
**H8**	H2	2.378	-0.022	x, y, z	x, 1/2-y, -1/2+z

Fig. S1

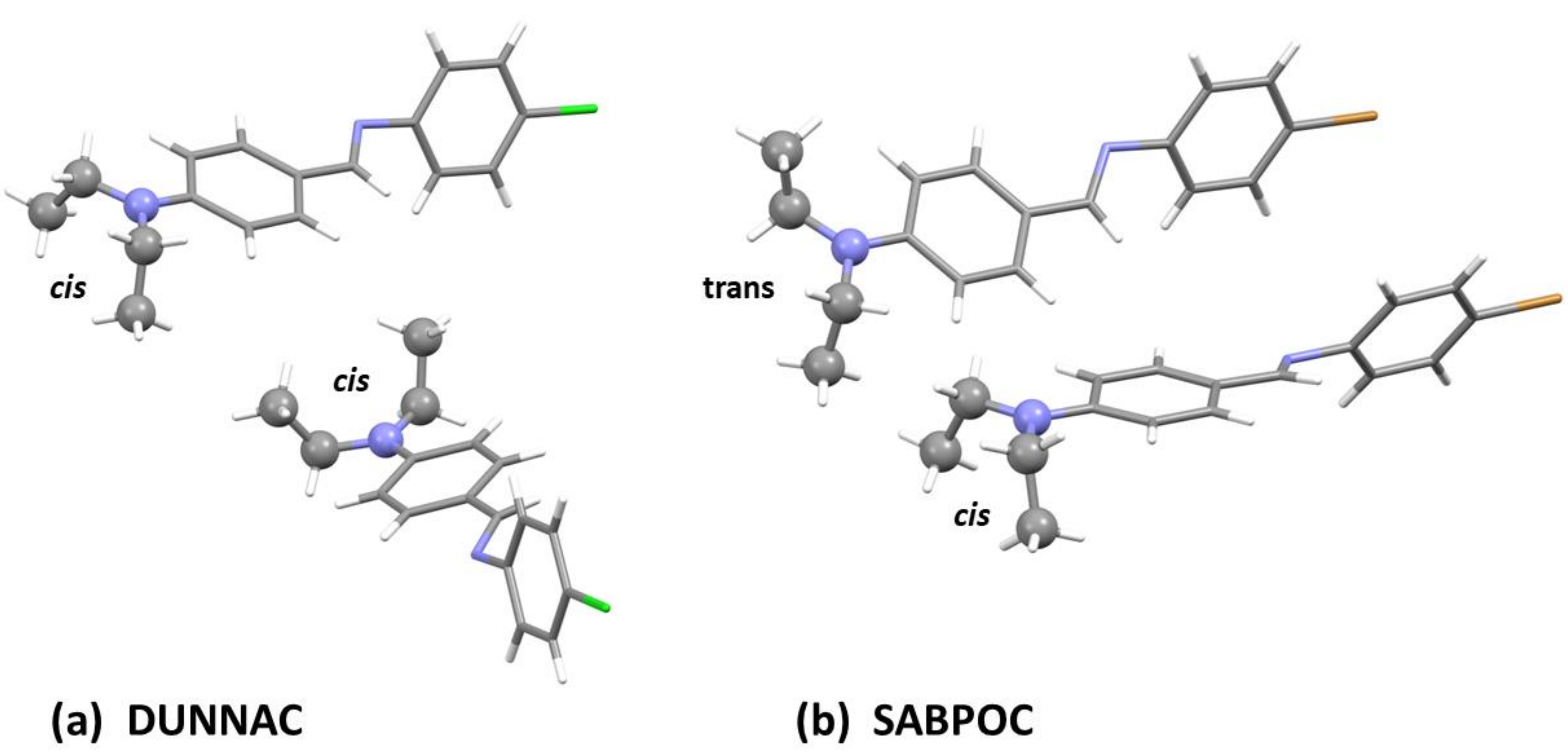

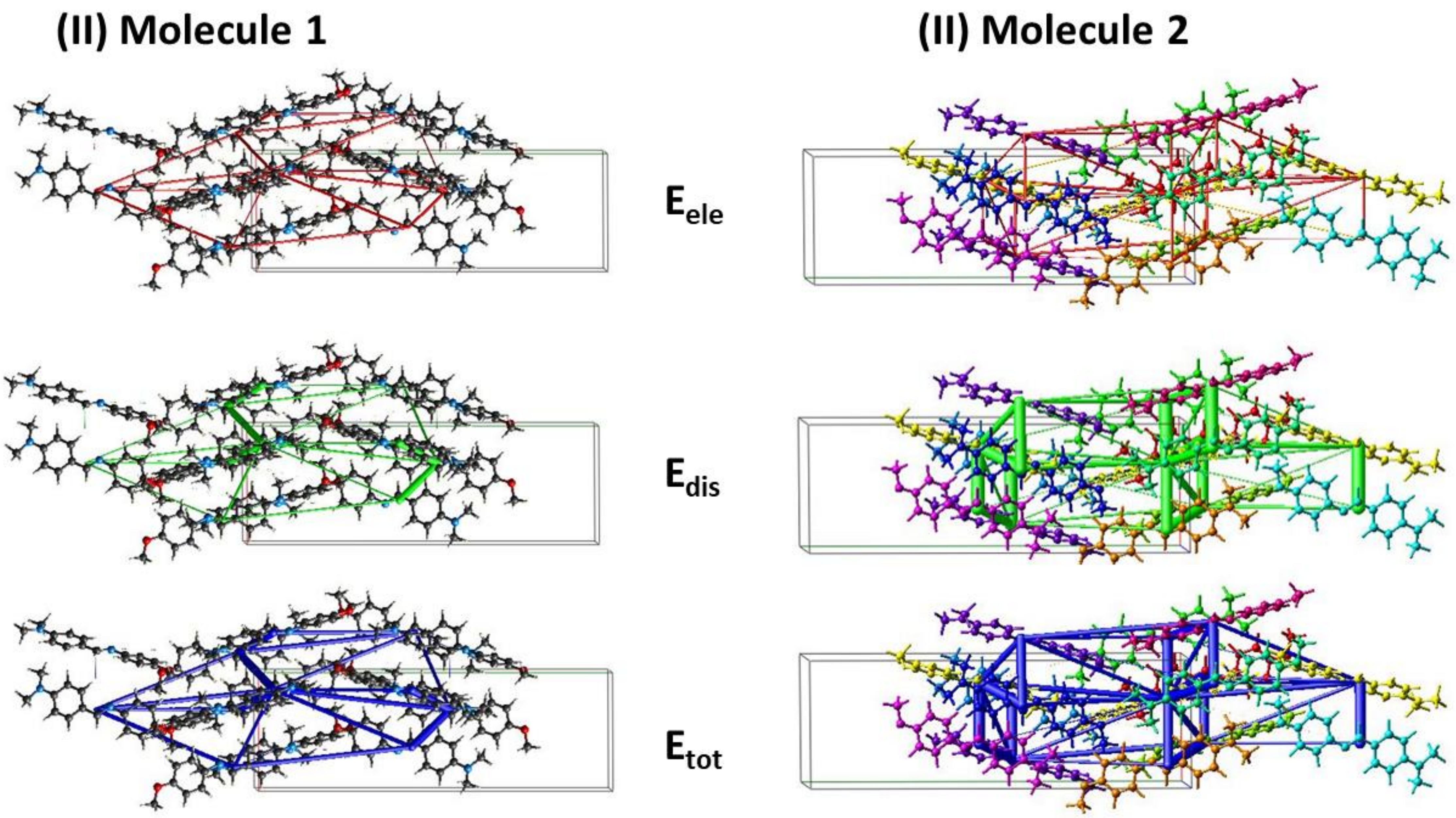

Fig. S3. Energy frameworks for molecules **1** and **2** of compound **II**.

(III)

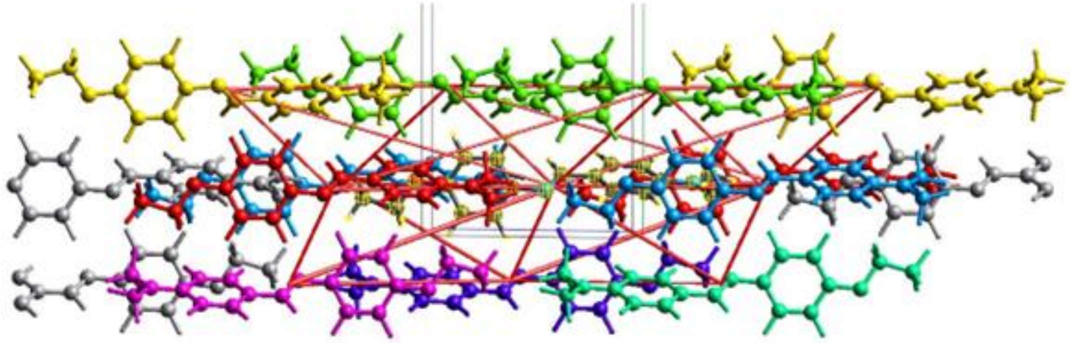

E_{ele}

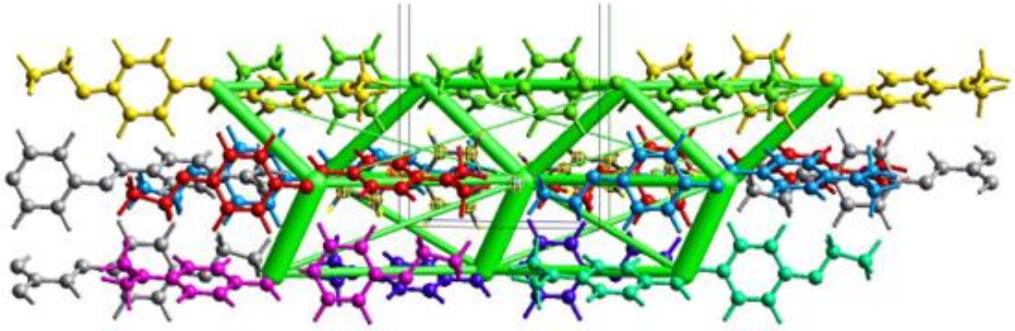

E_{dis}

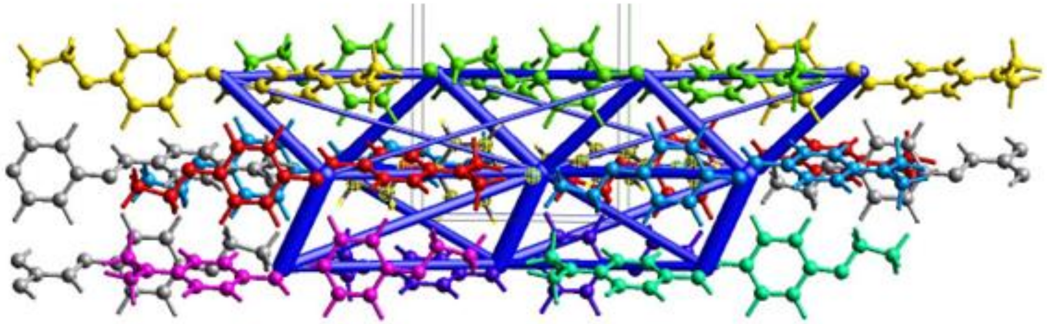

E_{tot}

Fig. S4 Energy frameworks for compound **III**

(a)

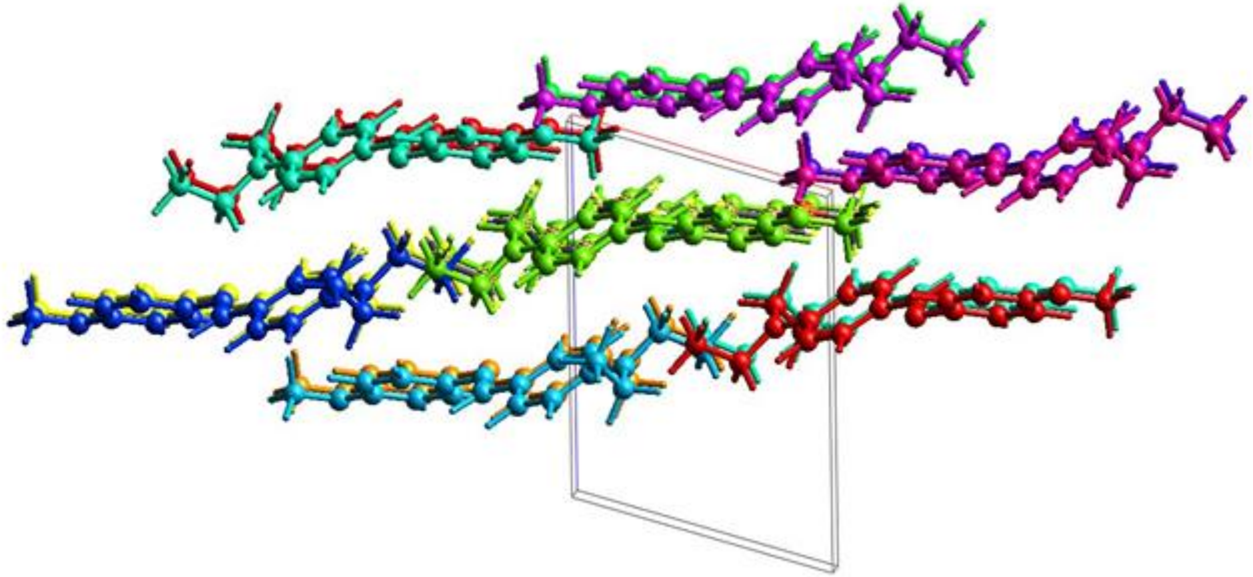

(b)

	N	Symop	R	Electron Density	E_ele	E_pol	E_dis	E_rep	E_tot
	2	x, y, z	14.11	HF/3-21G	-6.0	-1.7	-16.4	0.0	-22.0
	1	-x, -y, -z	10.28	HF/3-21G	-5.1	-2.2	-31.3	12.6	-24.7
	1	-x, -y, -z	16.03	HF/3-21G	13.3	-0.6	-16.2	0.0	-1.5
	2	x, y, z	9.29	HF/3-21G	-0.2	-0.8	-15.4	4.7	-10.8
	1	-x, -y, -z	5.87	HF/3-21G	-16.9	-5.5	-53.7	24.1	-49.7
	2	x, y, z	8.38	HF/3-21G	-11.4	-4.1	-41.4	19.6	-35.6
	1	-x, -y, -z	5.95	HF/3-21G	-20.8	-6.4	-49.5	22.9	-51.4
	1	-x, -y, -z	12.01	HF/3-21G	-6.7	-0.5	-14.0	0.0	-19.8
	1	-x, -y, -z	12.05	HF/3-21G	-4.6	-1.0	-15.8	0.0	-19.6
	1	-x, -y, -z	11.79	HF/3-21G	-0.4	-0.5	-8.4	2.0	-6.7
	1	-x, -y, -z	17.09	HF/3-21G	-2.8	-2.9	-10.8	0.0	-14.5

Fig. S5

(a) The colour-coded interaction mappings within a radius of 3.8 Å of a central reference molecule and
(b) the various contributions to the total energy (E_{tot}) for compound **I.**

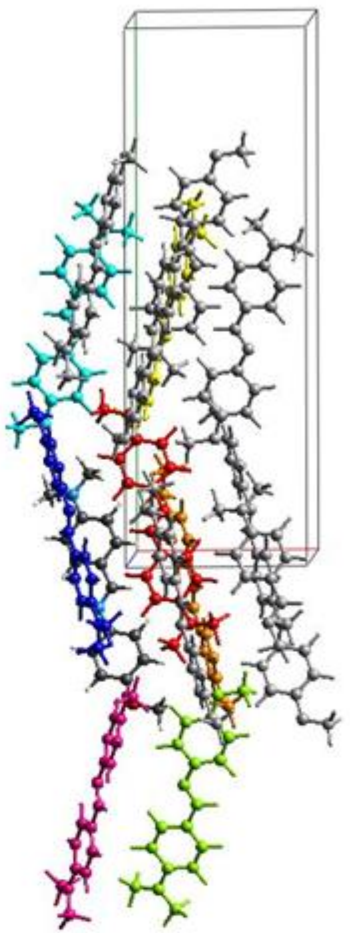

Molecule 1

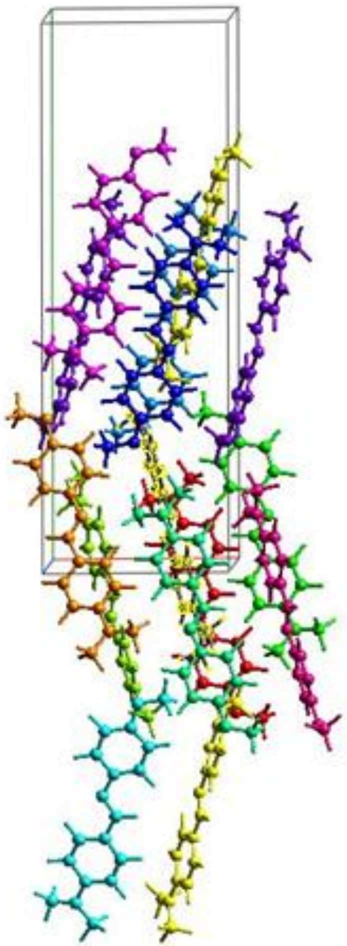

Molecule 2

Molecule 1

	N	Symop	R	Electron Density	E_ele	E_pol	E_dis	E_rep	E_tot
	1	-x, -y, -z	5.47	HF/3-21G	-13.7	-3.5	-36.5	13.7	-38.0
	1	-	4.73	HF/3-21G	0.0	nan	0.0	0.0	nan
	1	-	14.84	HF/3-21G	0.6	-0.0	-0.3	0.0	0.3
	1	x+1/2, -y+1/2, z+1/2	12.36	HF/3-21G	-12.0	-0.9	-8.3	0.0	-20.3
	0	-	11.75	HF/3-21G	-9.3	-0.9	-8.3	0.0	-17.5
	0	-	14.82	HF/3-21G	0.4	-0.0	-0.3	0.0	0.1
	1	-x+1/2, y+1/2, -z+1/2	14.49	HF/3-21G	-11.3	-1.9	-10.6	0.0	-22.3
	0	-	4.94	HF/3-21G	-0.3	-0.1	-1.2	0.0	-1.4
	1	-	5.27	HF/3-21G	-13.7	-3.5	-36.5	13.7	-38.0
	0	-x, -y, -z	9.52	HF/3-21G	-6.0	-1.2	-12.9	2.7	-16.2
	0	-	7.69	HF/3-21G	-0.8	-0.2	-2.8	0.0	-3.5
	1	-	11.67	HF/3-21G	0.3	-0.1	-0.9	0.0	-0.5

Molecule 2

	N	Symop	R	Electron Density	E_ele	E_pol	E_dis	E_rep	E_tot
	1	-	5.27	HF/3-21G	-13.4	-4.9	-50.4	22.8	-43.8
	1	-	4.94	HF/3-21G	-11.7	-4.6	-54.8	29.6	-40.3
	2	-x+1/2, y+1/2, -z+1/2	14.44	HF/3-21G	-12.2	-2.1	-12.5	0.0	-25.0
	1	-x, -y, -z	5.69	HF/3-21G	-7.2	-2.6	-33.2	12.7	-28.6
	1	-	4.73	HF/3-21G	-11.8	-6.2	-63.0	34.2	-45.2
	1	-	7.69	HF/3-21G	-11.8	-3.9	-42.3	18.7	-37.4
	1	-	14.82	HF/3-21G	5.9	-0.4	-5.7	0.0	0.6
	1	-	11.75	HF/3-21G	-6.5	-2.1	-17.9	14.3	-12.5
	1	-	11.67	HF/3-21G	-5.0	-1.9	-14.9	7.1	-14.1
	2	x+1/2, -y+1/2, z+1/2	12.38	HF/3-21G	-9.7	-0.9	-8.3	0.0	-17.9
	1	-	14.84	HF/3-21G	-1.3	-0.4	-3.2	0.0	-4.5
	1	-x, -y, -z	9.23	HF/3-21G	-8.3	-1.5	-14.4	3.4	-19.7

Fig. S6. Colour-coded interaction mapping within a radius of 3.8 Å of a central molecule and the various contributions to the total energy (E_{tot}) for compound **II**.

(a)

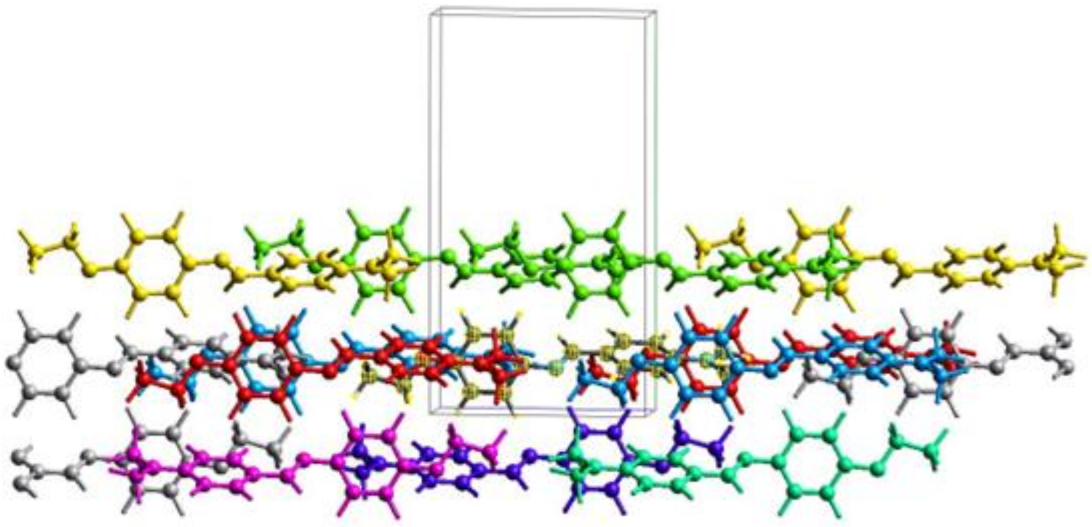

(b)

	N	Symop	R	Electron Density	E_ele	E_pol	E_dis	E_rep	E_tot
	2	x, y, z	11.17	HF/3-21G	-10.0	-2.5	-26.2	11.8	-25.9
	2	x, -y+1/2, z+1/2	15.14	HF/3-21G	-7.1	-0.5	-6.2	0.0	-13.2
	2	x, -y+1/2, z+1/2	6.38	HF/3-21G	-8.0	-3.5	-46.1	19.4	-36.3
	1	-x, -y, -z	8.84	HF/3-21G	-9.2	-3.2	-23.7	7.5	-26.7
	2	x, y, z	9.72	HF/3-21G	-5.1	-3.0	-31.3	12.8	-25.0
	1	-x, -y, -z	4.69	HF/3-21G	-8.7	-6.0	-69.8	31.0	-50.5
	1	-x, -y, -z	12.71	HF/3-21G	-12.6	-1.5	-17.7	0.0	-29.7

Fig. S7

(a) The colour-coded interaction mappings within a radius of 3.8 Å of a central reference molecule and
(b) the various contributions to the total energy (E_{tot}) of compound **III**.

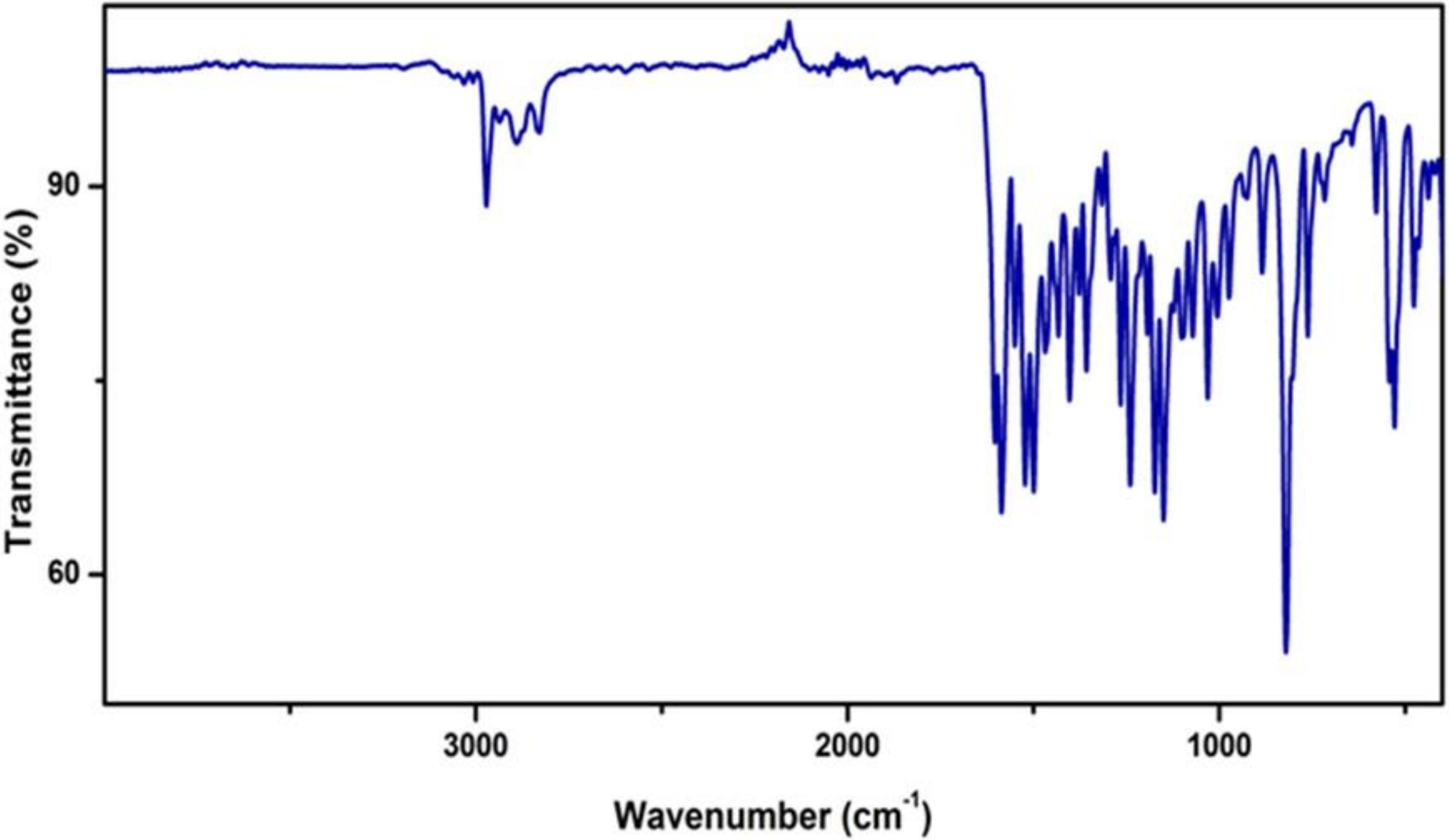

Fig. S8 IFTIR spectrum for compound **I**

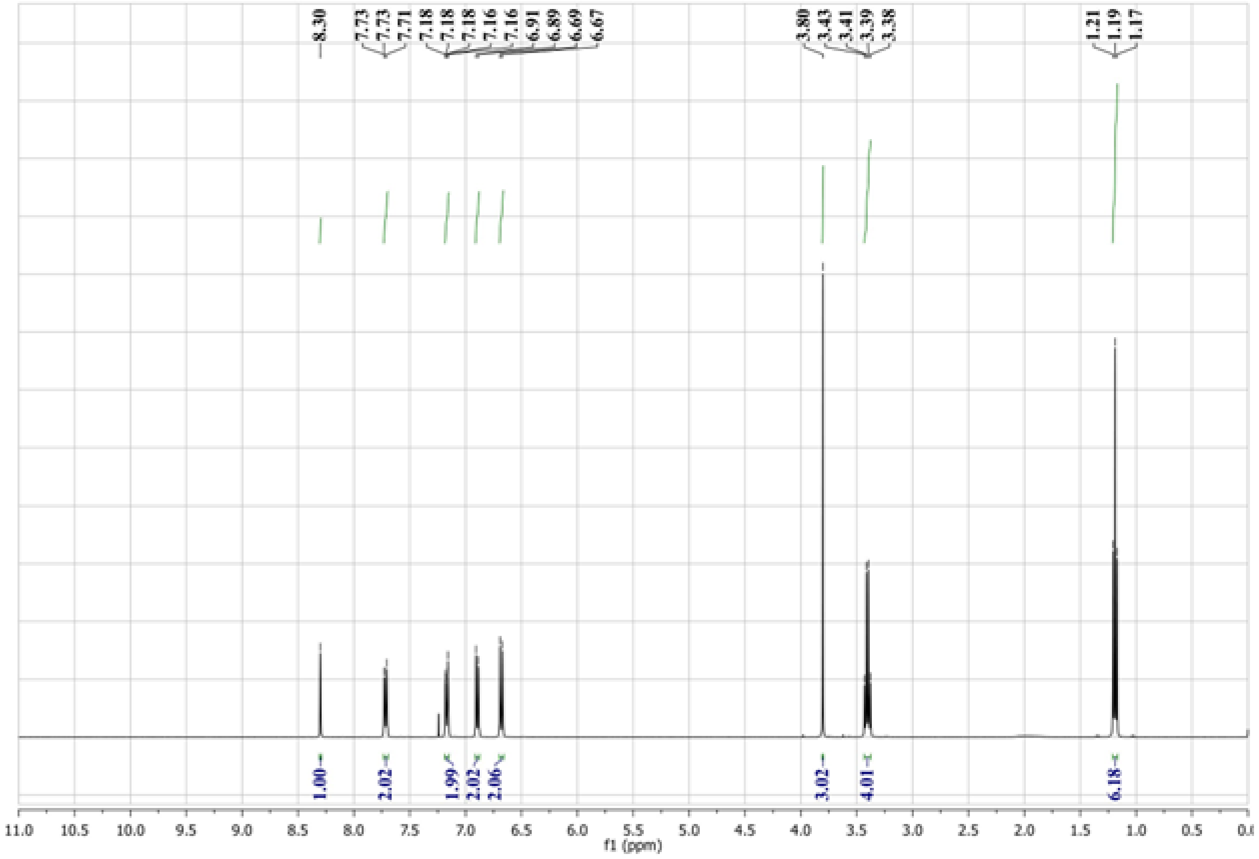

(a)

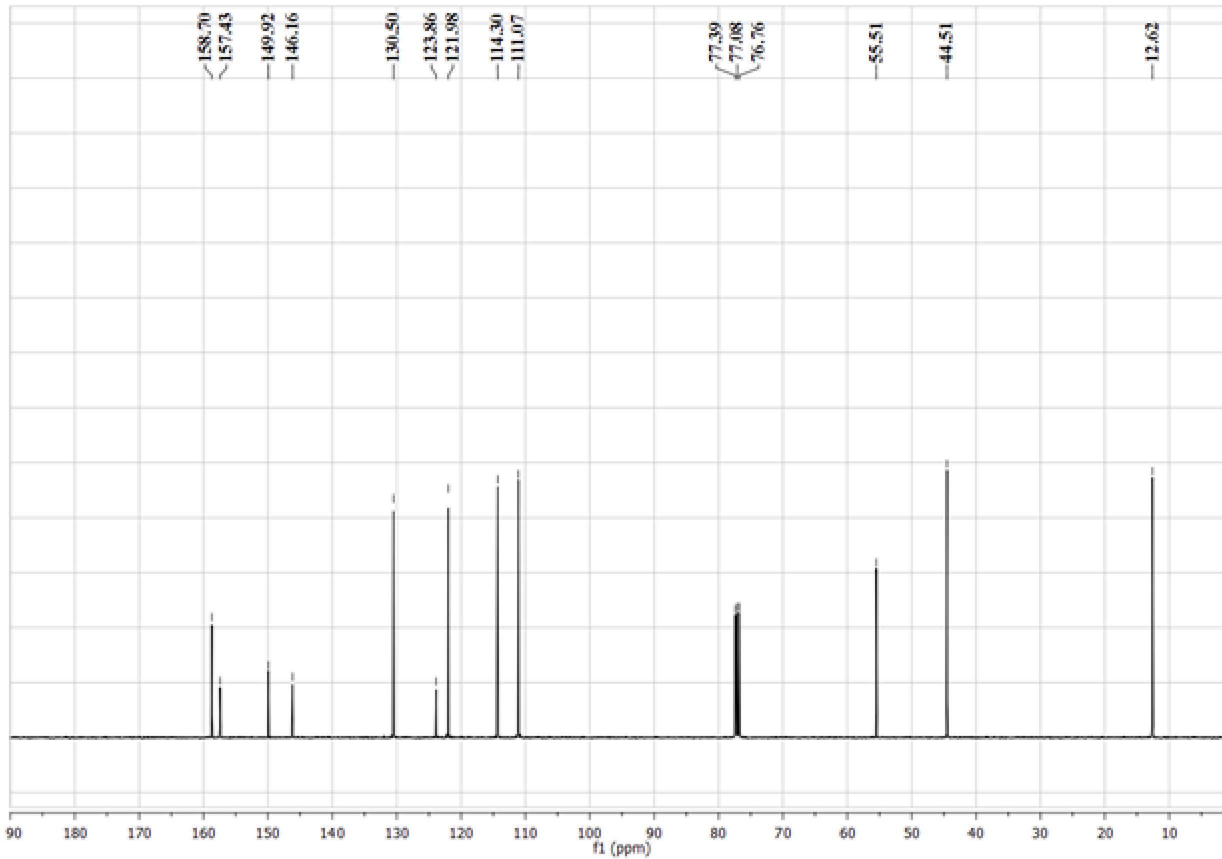

(b)

Fig. S9 (a) ^{1}H NMR spectrum for compound **I**, (b) ^{13}C NMR spectrum for compound **I**.

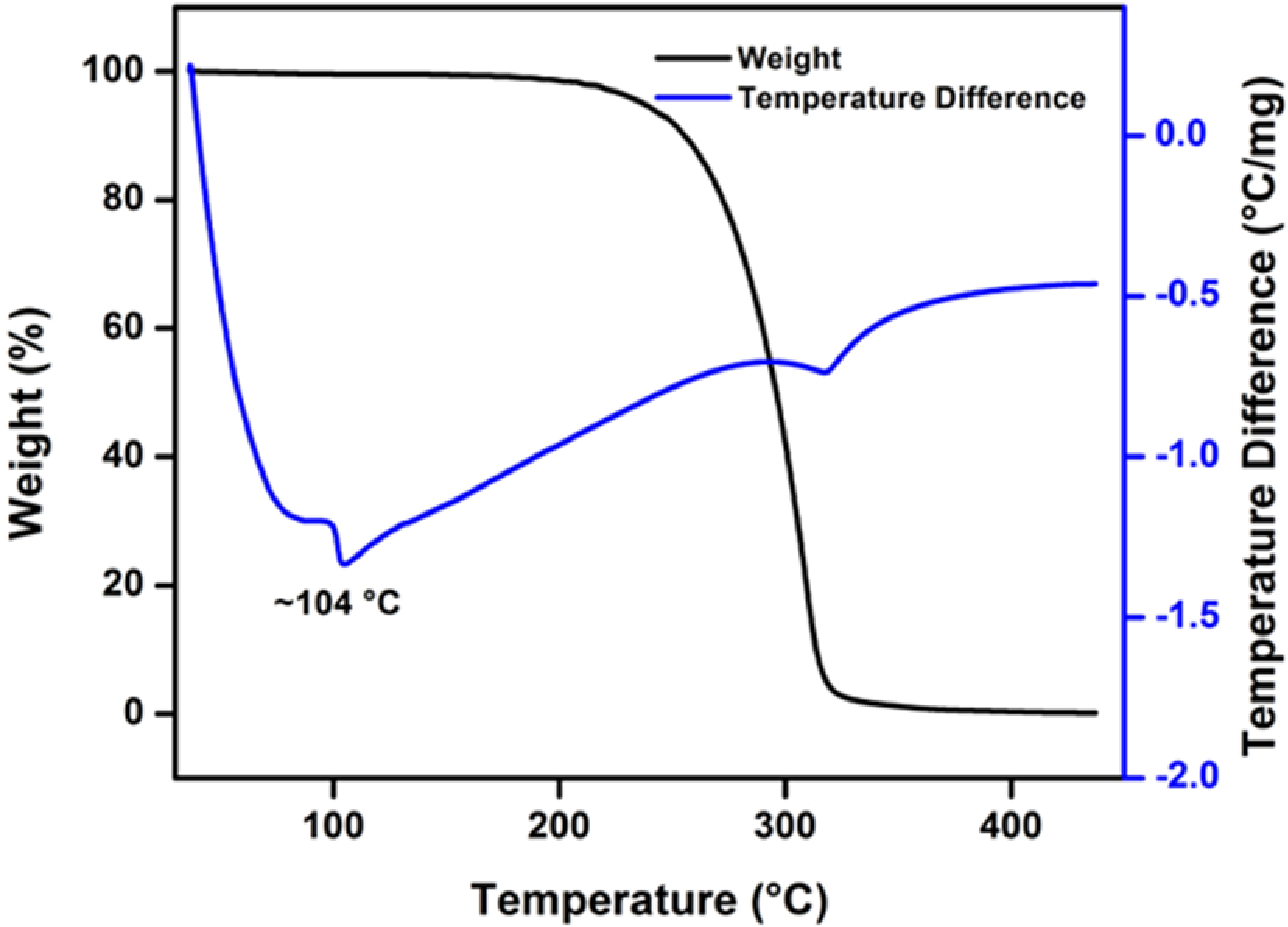

Fig. S10 Thermogravimetric analysis (TGA black) and differential thermal anlaysis (DTA blue) for compound **I**.
